# The association between paraoxonase 1 activity and the susceptibilities of diabetes mellitus, diabetic macroangiopathy and diabetic microangiopathy

**DOI:** 10.1111/jcmm.13711

**Published:** 2018-07-07

**Authors:** Diling Wu, Chenfang Wu, Yanjun Zhong

**Affiliations:** ^1^ ICU Center The Second Xiangya Hospital Central South University Changsha Hunan China

**Keywords:** activity, diabetes mellitus, meta‐analysis, paraoxonase 1

## Abstract

We carried out this meta‐analysis to explore the influence of paraoxonase 1 activity on the susceptibility of diabetes mellitus (DM), diabetic macroangiopathy and diabetic microangiopathy. Relevant studies were identified from PubMed, Web of Science and CNKI without language limitation, following the inclusion and exclusion criteria. Statistical analyses were implemented with the STATA 12.0 statistical software. Thirty‐six case‐control studies were included in the meta‐analyses, in which 35 for the association between paraoxonase 1 activity and DM risk, 8 for diabetic macroangiopathy and 7 for diabetic microangiopathy. Paraoxonase 1 activity was significantly associated with the susceptibility of DM in pooled population (SMD = −1.37, 95% CI = −1.79 ∼ −0.96, *P *=* *.000), and Asians (SMD = −2.00, 95% CI = −2.56 ∼ −1.44, *P *=* *.000), but not in non‐Asians (SMD = −0.44, 95% CI = −0.91 ∼ 0.03, *P *=* *.069). However, marked heterogeneity was existed (I^2^ = 98.10%, *P* = .000) and subgroup analyses failed to investigate the sources of heterogeneity. Then, meta‐regression was performed and found that ethnicity could explain the observed between‐study heterogeneity (*P *=* *.002). Meanwhile, significant associations were found between paraoxonase 1 activity and diabetic macroangiopathy (SMD = −1.06, 95% CI = −1.63 ∼ −0.48, *P *=* *.000) and diabetic microangiopathy (SMD = −0.72, 95% CI = −1.32 ∼ −0.13, *P *=* *.018). In conclusion, paraoxonase 1 activity plays important roles in the risk of DM, diabetic macroangiopathy and microangiopathy with ethnicity differences. Further studies with large sample and well design are needed to confirm these results.

## 1 INTRODUCTION

Diabetes mellitus (DM) is one of the most serious health‐related diseases all over the world, characterized by recurrent or persistent hyperglycaemia. It was estimated that approximately 90% DM were type 2 diabetes mellitus (T2DM), which occurs due to cells developing a resistance to insulin. DM prevalence is rapidly increasing in developing countries with the development of economics. When DM was not properly managed, it can lead to life‐threatening complications,^1^ including diabetic macroangiopathy (hear, brain, etc.,), diabetic microangiopathy (kidney, eye, etc.,) and neuropathy. DM is an important risk factor that leads to ischaemic stroke, and DM complicated with ischaemic stroke can aggravate the mortality and morbidity of patients.^2^, ^3^ Various factors contributed to the occurrence and development of DM, such as genetic, psychological, lifestyle and environment. Among these influential factors in DM, the serum level, the activity and gene polymorphisms of paraoxonase 1 played important roles in the susceptibility of DM.

Paraoxonase 1, a member of hydrolases with a glycoprotein structure, is a 354 aa glycoprotein of about 45 kD that is synthesized in the liver, released to the blood and binds to the high‐density lipoprotein in calcium‐dependent manner.^4^ Paraoxonase 1 has been demonstrated approximately 200 nucleotide polymorphisms, such as Q192R polymorphism, which was significantly associated with the susceptibility to T2DM by meta‐analysis.^5^ Paraoxonase 1 is a multifunctional enzyme with functions of arylesterase, paraoxonase and lactonase. Serum level and activity of paraoxonase 1 are different from individuals. Paraoxonase 1 activity is affected by age, sex, lifestyle and genetic polymorphisms.

It has been reported that paraoxonase 1 activity was decreased in heart disease,^6^, ^7^ and a large number of studies have been conducted over the last two decades to demonstrate the relationship of paraoxonase 1 activity and the susceptibility to DM and DM complications. Mackness et al^8^ firstly found that paraoxonase 1 activity was significantly higher in diabetic population than non‐diabetic population in 1998. While, Kopprasch et al. showed no significant difference in paraoxonase 1 activity between DM and non‐DM in different population.^9^, ^10^, ^11^, ^12^, ^13^, ^14^ Meanwhile, Mackness et al^15^ firstly described that paraoxonase 1 activity was significantly higher in DM patients without complications than that with retinopathy in 2000. Subsequently, there were some studies on the associations between paraoxonase 1 activity and the susceptibilities of DM and DM complications. However, the previously published results remain contentious. Therefore, to firmly demonstrate the association of paraoxonase 1 activity (paraoxon as substrate) with the susceptibility to DM, diabetic macroangiopathy and diabetic microangiopathy, we performed this meta‐analysis of data from thirty‐six studies.

## 2 METHODS

We carried out literature searches in PubMed, Web of Science and China National Knowledge Internet (CNKI) without language limitation, using the following key words: “PON1” or “paraoxonase 1,” “diabetes” or “diabetes mellitus” or “DM” or “T2DM” or “T1DM,” and “activity.” On the other hand, relevant articles were hand searched to identify additional reports.

### 2.1 Inclusion and exclusion criteria

The following criteria were used to select articles included in this meta‐analysis: (1) published as original case‐control studies; (2) paraoxonase 1 activity was detected in serum using paraoxon as the substrate; (3) reported an association between paraoxonase 1 activity and the susceptibilities of DM, diabetic macroangiopathy and/or diabetic microangiopathy. When the same series of patients were used in more than one article, we used the latest and most complete one.

### 2.2 Data extraction

WDL and WCF screened all searched articles and extracted data from all eligible publications independently. For each study, we carefully extracted the following information: name of the first author, published year, country, ethnicity, type of DM, type of control and paraoxonase 1 activity (mean ± SD) in each group. If there were any discrepancies, an agreement was reached after discussion; otherwise, another author was consulted to resolve the dispute.

### 2.3 Statistical analysis

Paraoxonase 1 activity in each group was described as mean ± SD. Standard mean differences (SMD) were used to evaluate the association between paraoxonase 1 activity and the susceptibilities of DM, diabetic macroangiopathy and/or diabetic microangiopathy. Heterogeneity was measured using a Chi‐square‐based *Q* test. The fixed effect model was used to calculated pooled SMD, when *I* ^2^ < 50% and *P *>* *.05. Otherwise, the random effect model was applied. Meta‐regression analyses on published year, ethnicity, sample size, type of control and type of DM, and subgroup analyses on type of control, ethnicity and type of DM were conducted to assess heterogeneity sources across the study. Publication bias was investigated using Egger's test and funnel plot. STATA 12.0 software (Stata corporation, TX, USA) was used in this meta‐analysis. *P *<* *.05 means statistical significance.

## 3 RESULTS

### 3.1 Study characteristics

**Figure 1:**
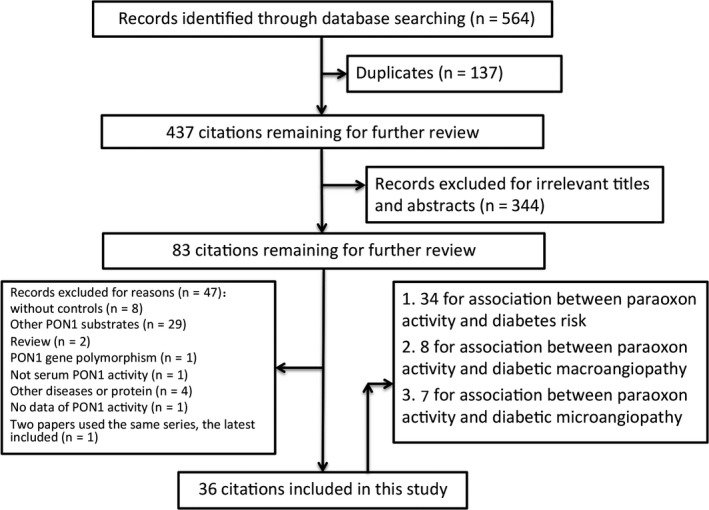
Flow diagram of the search strategy and study selection

Relevant publications were searched and preliminarily reviewed. As shown in Figure 1, 564 publications were identified, in which 137 duplicates and 344 irrelevant papers were excluded. Then eighty‐three citations remaining for further review. Eight original articles lacking of controls, twenty‐nine articles used other paraoxonase 1 substrates, two reviews, one performed not in serum, one with paraoxonase 1 gene polymorphism, four with other diseases or protein and one without data of paraoxonase 1 activity were all excluded. Two studies used the same case series, respectively, so the most recent publication was included.^8^, ^16^ Lastly, 36 case‐control studies were included in the meta‐analyses.^9^, ^10^, ^11^, ^12^, ^13^, ^14^, ^15^, ^16^, ^17^, ^18^, ^19^, ^20^, ^21^, ^22^, ^23^, ^24^, ^25^, ^26^, ^27^, ^28^, ^29^, ^30^, ^31^, ^32^, ^33^, ^34^, ^35^, ^36^, ^37^, ^38^, ^39^, ^40^, ^41^, ^42^, ^43^, ^44^ Thirty‐four publications were written in English, and two were written in Chinese.^17^, ^18^ The detailed information is shown in Tables 1 and 2. The first author, published year, country, ethnicity, the types and numbers of cases and controls, and levels of paraoxonase 1 activity for each study were presented.

**Table 1:** Characteristics of studies included in this meta‐analysis for the relationship between paraoxon activity and diabetes risk

Author	Year	Type of DM	Type of control	Country	Ethnicity	Paraoxon activity (DM)			Paraoxon activity (Control)		
						Mean^a^	SD	N	Mean^a^	SD	N
Inoue	2000	T2DM	Healthy control	Japan	Japanese	116	55	108	162	57	161
Mackness	2002	T1DM	Healthy control	English	Englishman	178.8^b^	71.23^b^	152	214.6^b^	99.08^b^	282
Letellier	2002	T2DM	Healthy control	France	French	217.63^b^	130.1^b^	167^b^	259^b^	154.3^b^	105
Kopprasch	2003	T2DM	Healthy control	Finland	Caucasian	5.33	0.5	75	4.67	0.27	403
Rosenblat	2006	NIDDM	Healthy control	Israel	Israelite	183	35	3	340	37	3
Mastorikou	2006	T2DM	Healthy control	English	Englishman	113.6^b^	92.3^b^	36	269.4^b^	104.75^b^	19
Singh	2007	T2DM	Healthy control	India	Indian	58.36	26.03	57	64.92	32.92	191
Flekac	2008	T2DM	Healthy control	Czech Republic	Czech	118	69	246	203	58	110
						110	68	86			
		T2DM									
Unur^c^	2008	T2DM	Healthy control	Turkey	Turk	135.7	71.1	51	364.2	26.7	53
Poh	2010	T2DM	Healthy control	Malaysia	Malaysian	486.31^b^	284.94^b^	140^b^	581	315	153
Abdin^c^	2010	T2DM	Healthy control	Egypt	Egyptian	18.79^b^	6.44^b^	40^b^	39.66	12.38	30
Nowak	2010	T1DM	Healthy control	Poland	Polish	266.83	164.94	76	312.04	129.77	35
Stefanovic	2010	T2DM	Healthy control	Serbia	Serbian	199.4	154.73	114	299.5	285.61	91
Altuner	2011	T2DM	Healthy control	Turkey	Turk	80.02^b^	12.94^b^	100^b^	94.12	6.79	50
Ergun	2011	T2DM	Healthy control	Turkey	Turk	134.67	66.99	171	244.45	114.2	80
Gelisgen^c^	2011	GDM	Without DM	Turkey	Turk	95.31	18.57	23	133.15	18.88	22
Gupta	2011	T2DM	Healthy control	Turkey	Turk	114.2^b^	47.38^b^	250	178^b^	63.58^b^	300
Liu	2011	T2DM	Healthy control	China	Chinese	142	55	100	222	99	50
Tabak^c^	2011	T2DM	Healthy control	Turkey	Turk	113.42	62.38	69	260.55	76	20
Ames	2012	T2DM	Healthy control	Mexico	Mexican	289.4	61.9	75	282.6	70.2	86
Gbandjaba^c^	2012	DM	Healthy control	Morocco	Moroccan	67.83	89.58	84	68.91	99.51	87
Guo	2012	T2DM	Healthy control	China	Chinese	102.24^b^	14.95^b^	108^b^	183.52	49.45	50
Murakami	2013	T2DM	Healthy control	Japan	Japanese	261	47	36	309	32	9
Bansal	2013A	T2DM	Healthy control	India	Indian	228.34^b^	37.83^b^	157^b^	379^b^	31.5^b^	40
Bansal	2013B	T2DM	Healthy control	India	Indian	209.7^b^	45.77^b^	265^b^	381^b^	30.33^b^	171
Budak	2013	T2DM	Healthy control	Turkey	Turk	210.81^b^	46.89^b^	54^b^	258	60	24
Helaly	2013	T2DM	Healthy control	Egypt	Egyptian	89.1	11.4	100	239.6	49.3	100
Fekih	2014	T1DM	Healthy control	Tunisia	Tunisian	330	198	122	334	220	97
Sreckovic	2014	GDM	Without DM	Austria	Austrian	35	9	9	75	18	11
Craciun	2016	T1DM	Healthy control	Romania	Romanian	181^b^	38.83^b^	82	215^b^	83.75^b^	41
Muhtaroglu	2016	DM	Healthy control	Turkey	Turk	73.15^b^	19.98^b^	60^b^	97.7^b^	23.175^b^	30
Shakeri	2017	T2DM	Healthy control	Iran	Iranian	770.95	320.47	90	2185.64	428.76	90
Sun	2017	DM	Without DM	China	Chinese	309.4^b^	116.8^b^	51	366.8^b^	120.8^b^	135
Crow	2018	T2DM	Without DM	America	American	51.1	35.5	117	52.4	32.9	117

DM, diabetes mellitus; SD, standard deviation; N, number; T2DM, type 2 diabetes mellitus; T1DM, type 1 diabetes mellitus; NIDDM, non‐independent diabetes mellitus; GDM, gestational diabetes mellitus.

a The unit of paraoxonase 1 activity is nmol/min/mL.

b Means calculated data from articles.

c The unit of paraoxonase 1 activity is U/mL.

**Table 2:** Characteristics of studies included in this meta‐analysis for the relationship between paraoxon activity and risk of diabetes complications

Author	Year	Type of DM	Country	Ethnicity	DM without complications			DM with macroangiopathy			DM with microangiopathy		
					Mean^a^	SD	N	Mean^a^	SD	N	Mean^a^	SD	N
Mackness	2000	T2DM	English	Englishman	164.1^b^	76.63^b^	93				113.4^b^	68.6^b^	101
Letellier	2002	T2DM	France	French	207^b^	132^b^	96	211^b^	189^b^	36	254^b^	155^b^	35
Abdin	2010	T2DM	Egypt	Egyptian	23.33	11.93	20				14.25	6.13	20
Nowak	2010	T1DM	Poland	Polish	289.87	157.07	35				227.66	123.57	41
Poh	2010	T2DM	Malaysia	Malaysian	583	342	44	442	244	96			
Liu	2011	T2DM	China	Chinese	152	67	50	110	46	50			
Guo	2012	T2DM	China	Chinese	125.14	34.22	29	94.49	28.87	37	93.26	26.81	42
Gupta	2012	T2DM	Turkey	Turk	114.2^b^	47.42^b^	250	51^b^	15.75^b^	300			
Bansal	2013A	T2DM	India	Indian	276^b^	49.75^b^	57	198^b^	35^b^	47	204	37.75^b^	53
Bansal	2013B	T2DM	India	Indian	246^b^	40.33^b^	135	172^b^	30.17^b^	130			
Budak	2013	T2DM	Turkey	Turk	221	52	29				199	39	25
Sun	2017	DM	China	Chinese	352	184	10	299	96	41			

DM, diabetes mellitus; SD, standard deviation; N, number; T2DM, type 2 diabetes mellitus; T1DM, type 1 diabetes mellitus.

a The unit of paraoxonase 1 activity is nmol/min/mL.

b Means calculated data from articles.

### 3.2 Paraoxonase 1 activity and DM risk

**Figure 2:**
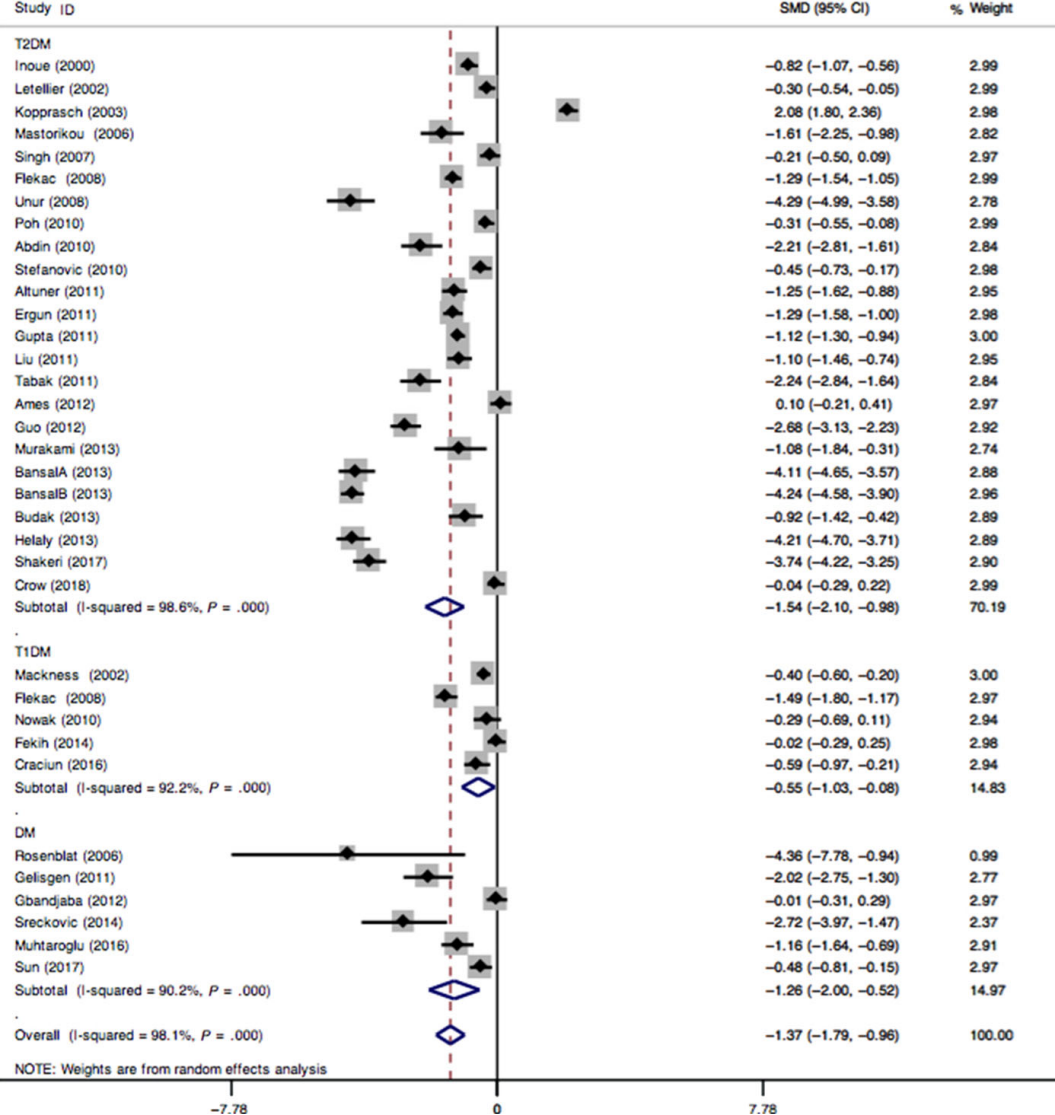
Forest plot for the association between paraoxonase 1 activity with DM, subgrouped by type of diabetes mellitus

As shown in Table 1, thirty‐four articles studied the association between paraoxonase 1 activity and risk of DM included 3474 DM patients and 3246 controls. Twenty‐one studies were performed in Asian countries,^10^, ^17^, ^18^, ^19^, ^20^, ^21^, ^22^, ^23^, ^24^, ^25^, ^26^, ^27^, ^28^, ^29^, ^30^, ^31^, ^32^, ^33^, ^34^, ^42^, ^43^ and other thirteen in non‐Asian.^9^, ^11^, ^12^, ^13^, ^14^, ^16^, ^35^, ^36^, ^37^, ^38^, ^39^, ^40^, ^41^ Twenty‐three articles were focused on T2DM, one on T1DM and T2DM,^37^ four on TDM,^13^, ^16^, ^38^, ^41^ two on GDM^26^, ^40^ or one on NIDDM,^20^ and three^12^, ^32^, ^34^ were unknown. The result showed significant heterogeneity was found (*I* ^2^ = 98.10%, *P *=* *.000, Figure 2). Therefore, we performed subgroup analyses on ethnicity (categorized as Asian and non‐Asian groups), type of DM (categorized as T2DM, T1DM and other DMs) and type of control (categorized as healthy control and without DM) to investigate the sources of heterogeneity. However, significant heterogeneities were still existed in each subgroup (data not shown). Therefore, meta‐regression was used to identify the sources of this heterogeneity, using the following covariates: published year, sample size, ethnicity, type of control and type of DM. The results showed that ethnicity could explain the observed between‐study heterogeneity (*P *=* *.002, Table 3).

**Table 3:** The meta‐regression results for the association between paraoxonase activity and susceptibility of diabetes mellitus

Covariates	Coefficient	Standard error	*Z* value	*P* value	95% confidence interval
Published year	−0.0329444	0.0581352	−0.57	.571	−0.1468872˜0.0809985
Sample size	0.0022293	0.0018531	1.20	.229	−0.0014027˜0.0058613
Ethnicity	−1.465915	0.4797128	−3.06	.002^a^	−2.406135˜−0.5256954
Type of control	−0.139538	1.085985	−0.13	.898	−2.268029˜1.988953
Type of diabetes mellitus	0.2003757	0.3617462	0.55	.580	−0.5086338˜0.9093852

a *P *<* *.05.

Meta‐analysis showed significant relationship between paraoxonase 1 activity and susceptibility of DM (SMD = −1.37, 95% CI = −1.79 ∼ −0.96, *P *=* *.000, Figure 2). Risk of DM obviously increased with low levels of paraoxonase 1 activity. Subgroup analysis on type of DM showed significant relationships found in T2DM subgroup (SMD = −1.54, 95% CI = −2.10 ∼ −0.98, *P *=* *.000), T1DM subgroup (SMD = −0.55, 95% CI = −1.03 ∼ −0.08, *P *=* *.023) and other types of DM (SMD = −1.26, 95% CI = −2.00 to −0.52, *P *=* *.001) (Figure 2). Meanwhile, the result of subgroup analysis on ethnicity showed significant associations between paraoxonase 1 activity and DM in Asian group (SMD = −2.00, 95% CI = −2.56 ∼ −1.44, *P *=* *.000, Supporting information Figure S1), but not in non‐Asian group (SMD = −0.44, 95% CI = −0.91 ∼ 0.03, *P *=* *.069, Supporting information Figure S1). Significant publication bias was found using funnel plot and Egger's test (*P *=* *.008, data not shown).

### 3.3 Paraoxonase 1 activity and susceptibility of diabetic macroangiopathy

**Figure 3:**
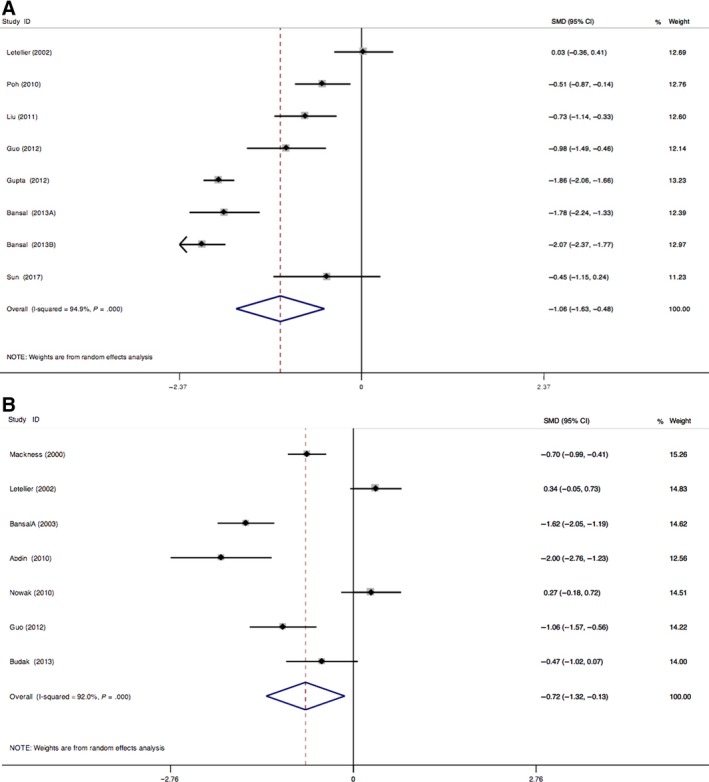
A, Forest plot for the association between paraoxonase 1 activity with diabetic macroangiopathy; B, Forest plot for the association between paraoxonase 1 activity with diabetic microangiopathy

As shown in Table 2, eight articles focused on the association of paraoxonase 1 activity and the risk of diabetic macroangiopathy, including 737 cases and 671 controls.^17^, ^18^, ^22^, ^30^, ^31^, ^34^, ^35^, ^44^ The results showed that significant heterogeneity was found (*I* ^2^ = 94.90%, *P *=* *.000, Figure 3A). Paraoxonase 1 activity was significantly associated with the susceptibility of diabetic macroangiopathy (SMD = −1.06, 95% CI = −1.63 ∼ −0.48, *P *=* *.000, Figure 3A). There was no significant publication bias existed, demonstrated by funnel plot and Egger's test (*P *=* *.116, data not shown).

### 3.4 Paraoxonase 1 activity and susceptibility of diabetic microangiopathy

As shown in Table 2, seven articles focused on the association of paraoxonase 1 activity and the risk of diabetic microangiopathy, including 317 cases and 359 controls.^15^, ^17^, ^23^, ^31^, ^35^, ^38^, ^43^ The results showed that significant heterogeneity was found (*I* ^2^ = 92.0%, *P *=* *.000, Figure 3B). Paraoxonase 1 activity was significantly associated with the susceptibility of diabetic microangiopathy (SMD = −0.72, 95% CI = −1.32 ∼ −0.13, *P *=* *.018, Figure 3B). Meanwhile, there was no significant publication bias, according to funnel plot and Egger's test (*P *=* *.533, data not shown).

## 4 DISCUSSION

Our meta‐analysis firstly demonstrated the associations between paraoxonase 1 activity and DM, diabetic macroangiopathy and diabetic microangiopathy by polling the individual data set. In the overall meta‐analysis of paraoxonase 1 activity, susceptibility of DM significantly increased with low levels of paraoxonase 1 activity, but strong between‐study heterogeneity was found. Therefore, to address the substantial heterogeneity, subgroup analyses on ethnicity, type of control and type of DM were conducted. But significant heterogeneity was still existed. Then, meta‐regression was performed using the following covariates: published year, sample size, ethnicity, type of control and type of DM and the results showed that ethnicity could explain the observed between‐study heterogeneity. In addition, subgroup analysis showed significant associations between paraoxonase 1 activity and DM in Asian group, but not in non‐Asian group. Paraoxonase 1 activity was different from ethnicity, and significantly higher in Malays and Chinese than Indians.^22^ A recent meta‐analysis on the associations of paraoxonase 1 Q192R/L55M genetic polymorphisms with susceptibility of T2DM also suggested a significant ethnicity differences.^5^ There are huge racial and regional differences in the genetic polymorphisms of paraoxonase 1, according to the 1000 genomes database.^5^ Genetic polymorphisms of paraoxonase 1 have been demonstrated to affect the paraoxonase 1 activity.^45^ Regarding the paraoxonase 1 Q192R polymorphism, paraoxonase 1 activity was different from genotypes, with the highest level in genotype RR in an Egyptian population,^42^ and the lowest level in genotype QQ in an African population.^46^ Paraoxonase 1 activity was significantly lower in the genotype QQ of paraoxonase 1 Q192R polymorphism than genotypes QR and RR in the patients with diabetic macroangiopathy.^42^ Meanwhile, paraoxonase 1 activity was significantly lower in the genotype LL of the paraoxonase 1 L55M polymorphism than genotypes LM and MM in the patients with diabetic macroangiopathy and microangiopathy in an Egyptian population, while no difference between L and M alleles.^42^ So the effect of interaction between paraoxonase 1 activity and genetic polymorphisms on the susceptibility of DM regarding the ethnic difference needs to be studied in the further.

Diabetic macroangiopathy is one of the most common complications of DM, which reduced the mortality and life quality of DM patients. It is a specific type of accelerated atherosclerosis. In this meta‐analysis, paraoxonase 1 activity was significantly associated with the susceptibility of diabetic macroangiopathy. Diabetic microangiopathy including diabetic nephropathy and diabetic retinopathy is also one of the most common complications. Diabetic nephropathy is the leading global cause of end‐stage renal disease. In this meta‐analysis, paraoxonase 1 activity was significantly associated with the susceptibility of diabetic microangiopathy.

Paraoxonase 1 can cleave oxidized lipids from low‐density lipoproteins. High‐density lipoproteins diminish the accumulation of lipid peroxides in low‐density lipoproteins mainly due to paraoxonase 1 activity. Paraoxonase 1 activity in human beings is very low at birth and increases with time.^47^ As our meta‐analysis has demonstrated the significant role of paraoxonase 1 activity in DM and diabetic complications, therapeutic strategies targeting this enzyme maybe reasonable. Therapeutic strategies can be focused on lifestyle modification and increasing the level of paraoxonase 1 activity.

In this meta‐analysis, there existed some limitations. First, only articles with English and Chinese language were included in this study, some publication bias may exist. Second, significant heterogeneity was found in our analysis about the association between paraoxonase 1 activity and susceptibility of DM and diabetic complications, and for DM, ethnicity might explain some of the sources of between‐study heterogeneity. But there were only 8 and 7 articles included for analysis of the association between paraoxonase 1 activity and diabetic macroangiopathy and microangiopathy, respectively, so we failed to find the sources of heterogeneity. Thus, large sample and well‐designed studies needed to demonstrate the result in the further.

In conclusion, paraoxonase 1 activity plays important roles in the risk of DM, diabetic macroangiopathy and microangiopathy with ethnicity differences. Further studies with large sample and well design are needed to confirm these results.

## CONFLICT OF INTEREST

All authors declared that there were no potential conflict of interests.
