# Supplementary figures and images for: The association between paraoxonase 1 activity and the susceptibilities of diabetes mellitus, diabetic macroangiopathy and diabetic microangiopathy
Source: J Cell Mol Med. 2018 Jul 7;22(9):4283–91. doi: 10.1111/jcmm.13711 (PMC6111876; doi:10.1111/jcmm.13711)

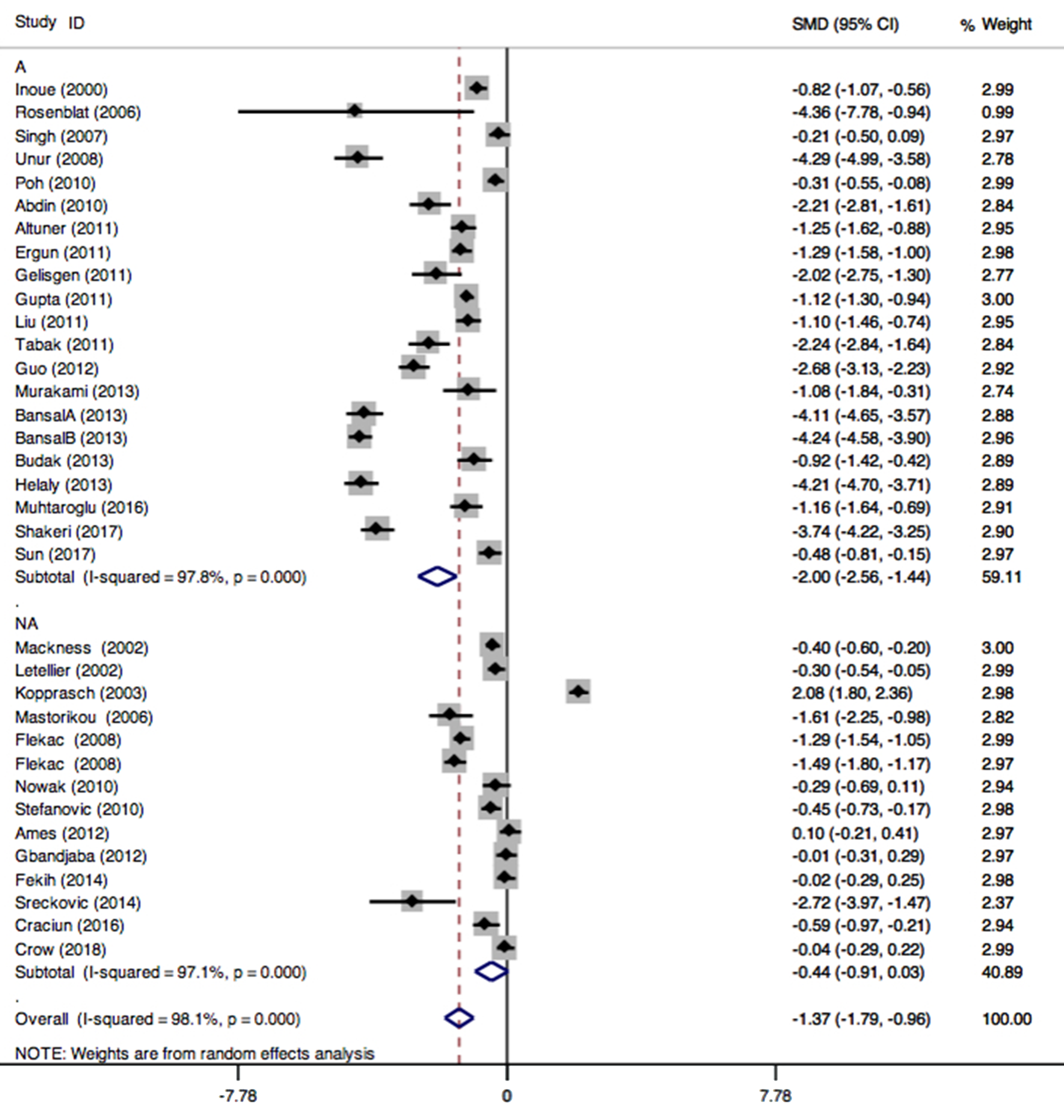

Supplement: Supplementary file 1 [file JCMM-22-4283-s001.tif]
